# Book Review

**DOI:** 10.1120/jacmp.v3i1.2593

**Published:** 2002-01-01

**Authors:** Steve P. Lee

**Affiliations:** ^1^ Department of Radiation Oncology B265 200 UCLA Medical Plaza Los Angeles CA 90095‐6951

## Abstract

**Applied Radiobiology and Bioeffect Planning.** David R. Wigg, Medical Publishing, Madison, WI, 2001.

PACS number(s): 87.50.–a

Since its inception a century ago, radiation therapy has always been done with meticulous quantification of dosage deposited within a patient's body. With the development of a modern treatment planning system, the spatial dose distributions are readily available in three‐dimensional displays. Nevertheless, conventional treatment plans based on such physical dosimetry alone ignore many biological considerations that impact directly on clinical results. One noted issue comes from the realization that some biological tissues (the so‐called late‐responding tissues) are much more sensitive to the variation in fractionation size than others (the acute‐responding tissues).^1^ Applied to clinical practice, this added complexity due to such radiobiology consideration has been described as the “double‐trouble” effect by Withers,^2^ i.e., the heterogeneity in total dose, as well as in the dose per fraction, would magnify the biologic consequence hidden inconspicuously within physical dosimetry. Conscientious clinicians often estimate such biological effect qualitatively and decide upon a particular treatment plan accordingly. However, an explicit representation of biologically oriented treatment plan, if feasible at all, would be more precise quantitatively, and thus, represents a useful clinical tool.

This book by D. R. Wigg attempts, therefore, to provide a massive collection of mathematical models used throughout the history of radiation therapy, specifically addressing the issue of biologically oriented dosimetry–termed “bioeffect planning” by the author. It is impressive in the scope of topics covered, as well as the depth of algebraic derivations developed largely by the author himself. I would not repeat the task of providing a chapter by chapter summary, which has been done succinctly in the Forewords by A. H. Beddoe, and Chapter 1, Sec. 1.1 by the author. Instead, I would like to point out some of its strengths, as well as what I personally feel as drawbacks, for the general readership of the Journal of Applied Clinical Medical Physics.

The first major strength is the comprehensive coverage of clinical radiation biology. Specifically, all relevant clinical issues are addressed, such as those pertaining to time, dose, fractionation, and volume effect. It is also comprehensive in its coverage of the historical development of quantitative radiobiology, dating back to the times when empirical power laws (e.g., concepts of NSD, TDF, and CRE) dominated. Nevertheless, I feel that the author dwells perhaps too much upon these archaic mathematical models that have been ruled obsolete by most radiation biologists and oncologists.^3^ There is a sense of excessive personal indulgence by the author of the materials that are outdated, and perhaps downright false in the science that they once represented. The detailed algebraic manipulations of these theories in their most elaborated form is a luxurious feast for anyone interested in the history of quantitative radiobiology, but may be misleading for oncologists or physicists searching for useful guidelines in patient care.

Fortunately, the author does provide coverage of the widely accepted radiobiology models centered largely upon the linear‐quadratic (LQ) formulation. The scientific validity of the LQ model has emerged as a result of the progress made in radiobiological investigations over the last few decades. The extension of the theory as covered in this monograph serves as a useful supplement to classic texts such as Thames and Hendry.^4^ In my mind, this is perhaps the most useful aspect of this book, especially when equipped with the “plausible parameter values” for the model as provided in Chapter 8. The author, however, seems to omit rigorous discussion about the theoretical foundation of this useful and popular model. For example, the repair factors in the *important incomplete* repair model of Thames are presented [Eqs. (3.8) and (4.4)] without formal step by step derivation (although one can obtain that from the textbook quoted above). An in‐depth discussion upon the hypothesized “mechanistic” or “biophysical” interpretation of the LQ parameters could also be beneficial for readers unaware of the controversy involved.^5^ ^,^ ^6^ Finally, the book neglects to discuss in detail the concept of the “biologically effective dose” (BED) which is gaining popularity in bioeffect planning,^7^ ^,^ ^8^ although the original term used by Barendsen (extrapolated response dose) is mentioned very briefly. In fact, the term BED is not even listed in the Index. This negligence is rather inexcusable due to the broad scope of subjects the whole monograph attempts to cover, and perhaps reflects the author's personal bias towards selective topics.

Perhaps one of the most important pieces of information readers of this book would wish to obtain is the mathematical formalism for the so‐called “volume effect,” as precision‐oriented treatment techniques such as conformal therapy and intensity modulated radiation therapy are gaining momentum in clinical implementation and now require desperately practical guidelines for their “biological optimization.” Unfortunately, the reader may be disappointed to find that Chap. 2 of this book entitled, “The Volume Effect,” fails to present the most updated development in this area. Instead, it draws attention to empirical formulations based on power laws and rudimentary assumptions about the geometrical relationships among length area, and volume. To defend such action, the author argues that the modern treatment approach using dose‐volume histogram analysis is of limited use primarily due to its complexity. The counterpoint is that the author's own mathematical expositions are perhaps even more difficult to understand by average clinical oncologists or physicists, due to the many arbitrary parameters introduced empirically. Furthermore, the author states at the very beginning of this book, “all models are wrong, but some are useful.” A personal corollary of mine is, while no model can ever be proven right, those that are indeed proven wrong are not useful. In such regard, the author's tendency to revive the already‐proven wrong models of yesterday serves no enlightened soul of today.

In summary, the major strengths of this book are the comprehensive scope of subjects covered and the extent of the author's laborious attempt to carry algebraic derivation to full details, coupled by examples using graphic illustrations. The author is to be congratulated for this diligent effort in producing such a monumental work, which represents probably a lifelong worth of scholarly pursuit. On the other hand, I feel that the discussion on archaic theories like power laws is overdone and may even be detrimental for pedagogical purpose. The theoretical development for currently accepted models including the LQ formalism is lacking in rigor (though available in literature elsewhere), but the discussion on the useful ranges of various biological parameters specified in the models is quite helpful. Overall, this book serves as a useful but nonessential addition to the library collection of radiation oncologists, biologists, and physicists who wish to further enhance their knowledge in the use of clinically relevant mathematical models.
